# Insights into Modern Therapeutic Approaches in Pediatric Acute Leukemias

**DOI:** 10.3390/cells11010139

**Published:** 2022-01-02

**Authors:** Kinga Panuciak, Mikołaj Margas, Karolina Makowska, Monika Lejman

**Affiliations:** 1 Student Scientific Society, Laboratory of Genetic Diagnostics, Medical University of Lublin, 20-093 Lublin, Poland; kinga.panuciak26@gmail.com (K.P.); mikolajmargas@interia.pl (M.M.); makowska.karolinaa@gmail.com (K.M.); 2 Laboratory of Genetic Diagnostics, Medical University of Lublin, 20-093 Lublin, Poland

**Keywords:** immunotherapy, CAR-T, antibodies, immune checkpoint inhibitors, PRR, pediatric leukemias

## Abstract

Pediatric cancers predominantly constitute lymphomas and leukemias. Recently, our knowledge and awareness about genetic diversities, and their consequences in these diseases, have greatly expanded. Modern solutions are focused on mobilizing and impacting a patient’s immune system. Strategies to stimulate the immune system, to prime an antitumor response, are of intense interest. Amid those types of therapies are chimeric antigen receptor T (CAR-T) cells, bispecific antibodies, and antibody–drug conjugates (ADC), which have already been approved in the treatment of acute lymphoblastic leukemia (ALL)/acute myeloid leukemia (AML). In addition, immune checkpoint inhibitors (ICIs), the pattern recognition receptors (PRRs), i.e., NOD-like receptors (NLRs), Toll-like receptors (TLRs), and several kinds of therapy antibodies are well on their way to showing significant benefits for patients with these diseases. This review summarizes the current knowledge of modern methods used in selected pediatric malignancies and presents therapies that may hold promise for the future.

## 1. Introduction

Acute leukemias are the most frequent cancer in children. The most common is acute lymphoblastic leukemia (ALL), which accounts for over 80% of all cases of acute leukemia. ALL cases are classified as B-ALL or T-ALL, based on immunophenotyping, with B-ALL encompassing approximately 85% of cases. In the 1960s, the five-year survival rate for pediatric patients with ALL was under 10%, while now, the survival of children treated in high-income countries exceeds 90% [1,2,3]. Nowadays, improvement in outcomes with conventional chemotherapy is challenging due to adverse effects increasing during further intensification of chemotherapy. Based on genome-wide analyses, B-ALL can be classified into more than 30 B-lineage subtypes with prognostic and therapeutic implications. The most important of them are presented in Table 1. Accurate identification of the genetic abnormalities in ALL is important to risk-stratify the relapse and to guide the incorporation of molecular targeted therapeutic approaches to reduce the risk of relapse. 

T-cell acute lymphoblastic leukemia (T-ALL) accounts for 10–15% of childhood leukemias and is characterized by a wide spectrum of genetic diversity, including chromosomal translocations that affect gene expression, the deregulation of oncogenes, submicroscopic deletions, disordered kinase signaling and cell cycles, and ribosomal dysfunction. Oncogenic transcription factors can be divided into several groups: *TAL1/2*, *LMO1*/2, *TLX1*/3, *LYL*, *HOXA-*translocations, *MEF2C*, *NKX1/2*, *CALM::AF10*, and *KMT2A*-rearrangements (*KMT2A*-r). Classical genetic mutations, identified in T-ALL by sequencing, include *NOTCH1*, *FBXW7*, *CDKN2A/2B*, *PHF6*, *USP7*, *PTEN*, *DNM2*, and *BCL11B* [19]. The genetic alterations in T-ALL are diverse, and no clear associations with outcomes have yet been identified.

Childhood acute myeloid leukemia (AML) is a genetically heterogeneous group of myeloid malignancies that account for approximately 20% of pediatric leukemias, with a median age of 6 years [20]. Pediatric AML has better outcomes than adults because of the more frequent presence of good prognostic genetic features and a higher tolerance of intensive treatment. WHO classification of AML distinguishes over 20 different subtypes of AML, defined based on distinct combinations of clinical, prognostic, morphologic, immunophenotypic, and genetic data [21]. The latter type of data is presented in Table 2.

The described genetic changes in pediatric AML have also become the starting point for new therapeutic strategies, some of which have already been approved by the US Food and Drug Administration (FDA). Among them are: FLT3 inhibitors, DOT1L inhibition, and epigenetic modulators.

FLT3 is a type III transmembrane receptor tyrosine kinase, whose role is to regulate hematopoiesis through the phosphorylation of, e.g., STAT5 and the activation of oncogenic pathways such as Ras/Raf/MAPK and PI3K/Akt/mTOR. Thus, FLT3 activation leads to decreased maturation and increased proliferation of bone marrow (BM) progenitor cells, making patients with FLT3 eligible for high risk. Therefore, it is advantageous to use inhibitors of this kinase. The best known of them is sorafenib, which has shown significant efficacy in relapsed/refractory (R/R) pediatric AML. In addition to sorafenib, the group of FLT3 inhibitors also includes sunitinib, lestaurtinib, and midostaurin. Currently, studies are underway on the next generations of drugs from this group (quizartinib, crenolanib, gilteritinib), which, due to their greater specificity towards FLT3, are characterized by a stronger inhibition of FLT3 [22].

The most common genetic disorders in pediatric AML are rearrangements of the *KMT2A* (formerly *MLL*) gene. *KMT2A* is a nuclear protein that regulates gene expression by catalyzing the methylation of lysine 4 on histone 3, and it is, therefore, critical to hematopoietic development. Studies show that the histone methyltransferase disruptor in telomeric silencing type 1 (DOT1L) is involved in promoting cell proliferation with *MLL* rearrangement, thereby promoting expression of leukemic genes. Therefore, it has become beneficial to develop inhibitors of DOT1L. Although one of them, pinometostat, as a standalone therapy, brought limited benefit in clinical trials, it has been proven that its initial treatment sensitizes AML cell lines to further sorafenib treatment. Moreover, recent studies also show a role for DOT1L in leukemia without *KMT2A* rearrangement. By comparing the consequences of DOT1L inhibition in both AML cells with and without *MLL* rearrangement, Lonetti et al. showed that pinometostat-mediated cytotoxicity is not closely related to *KMT2A* fusions [29].

As the common feature of AML is an altered epigenetic pattern, the Bromodomain family of proteins and the extracorporeal domain (including the proteins BRD2, BRD3, BRD4, and BRDT) have recently been identified. The role of these proteins is to regulate gene transcription by interacting with acetylated histones, thereby facilitating the activation of transcription. Therefore, they have become an important epigenetic target, and their inhibitors have shown anti-leukemic activity in preclinical models. Currently, they are being tested in the context of adult AML patients, which suggests the possibility of their use in pediatric AML [22].

This review will summarize the latest advances in the treatment of leukemia, focused on mobilizing and impacting a patient’s immune system. Below, we describe the latest developments in individual areas of leukemia immunotherapy.

## 2. FDA-Approved Drugs to Treat ALL or AML

### 2.1. CAR-T Immunotherapy—Genetically Modified T Lymphocytes 

Chimeric antigen receptor (CAR) is an unnatural form that, after activation in T-cells, imitates their receptor and leads to their effective function towards a precise antigen.

#### 2.1.1. Chimeric Antigen Receptor

CAR contains four separate modules: the antigen recognition domain, the extracellular hinge region, the transmembrane domain, and one or more intracellular T-cell signaling domains. An antigen recognition domain of the single-chain variable fragment (scFv) is a chimeric protein. It is composed of both light and heavy immunoglobulin chains that are linked together by a peptide linker [39]. The variant of the selected heavy and light immunoglobulin chains depends on their ability to bind to a target antigen, such as CD19. The linker is made of hydrophilic residues containing sections of serine and glycine (affecting flexibility) and sections of glutamate and lysine (providing additional solubility) [40]. In addition to scFv, the following have also been successfully used to target the specificity of CAR: cytokines, growth factors, innate immunity receptors, and compounds from the tumor necrosis factor (TNF) receptor superfamily [41]. The hinge region, also known as a spacer, is a small structural domain. It is located between the antigen-recognition domain and the outer membrane of the cell. Its role is to increase the flexibility of the scFv receptor head, leading to a decrease in the distance between CAR and its target antigen. Spacer building sequences are usually based on the proximal regions of membrane molecules such as IgG, CD8, and CD28 [41,42]. The transmembrane domain is made up of a hydrophobic alpha helix that encompasses the cell membrane. Its function is to anchor CAR to the plasma membrane, thereby connecting the extracellular hinge and antigen recognition domains with the intracellular signaling region. It ensures the stability of the entire receptor. The intracellular signaling domain of T cells is located in the endodomain of the receptor [41]. When an antigen is bound to an external antigen-recognition domain, CAR receptors aggregate and transmit an activation signal. Due to the inner cytoplasmic end of the receptor, it is then fixed inside the cell [39]. Natural activation of the T lymphocyte is based on the phosphorylation of immunoreceptor tyrosine-based activation motifs (ITAMs), which are present in the cytoplasmic domain CD3-ζ. To imitate this process, this domain is also commonly used in CAR as a main endodomain component [43]. To persist after activation, apart from signaling CD3, T lymphocytes also need costimulatory molecules. For this reason, CAR endodomains contain chimeric domains from costimulatory proteins. So far, the CD28, CD27, CD134 (OX40), and CD137 (4-1BB) have performed well in this role [41]. Their use significantly improves T cell proliferation, cytokine secretion, resistance to apoptosis, and in vivo stability [44]. There is a complicated relationship between the domains that make up CAR, and there is, therefore, no single optimal configuration. The exact composition of the used CAR remains largely empirical and depends on an analysis of tumor recognition in vitro [45,46].

#### 2.1.2. Vectors

The genetically engineered CAR fusion protein is transduced into the autologous T-cell by means of a retrovirus or lentivirus [45]. By partially deleting the U3 region of the 3′ long terminal repeat (LTR), the transcriptional activity of the virus is decreased significantly [47]. Although they have become safer, there is still a risk of uncontrolled integration leading to the overexpression of neighboring genes or the disruption of genes at the site of integration. To prevent this, non-viral plasmids (pEPI series) containing a scaffold/matrix attachment region (S/MAR) element have been developed for the episomal long-term expression of transgenes [48]. S/MAR is a segment of genomic DNA whose role is to anchor chromatin in nuclear matrix proteins and mediate the structural organization of chromatin in the nucleus. It binds to the protein A of the scaffold attachment factor (SAF-A) and thus ensures mitotic stability of the plasmids. Jin et al. have confirmed that non-integrating lentiviral vector (NILV) containing S/MAR may become the optimal vector with a low risk of insertional mutagenesis with long-term expression of the transgene [49].

Apart from the effectiveness and safety of the used vectors, the possibilities of their production are also important. Until now, their production involved a relatively long period of time and many stages, which significantly increased the costs of the entire process. As a solution, non-viral allogeneic engineering of the T-cell population, according to a cytokine-induced killer cell (CIK) differentiation protocol may be used here [50]. They are characterized by enrichment in CD3+ and CD56+ cytotoxic cells, which ensures high safety and minimal risk of graft versus host disease (GvHD) after allogeneic CIK. Additionally, by using Sleeping Beauty (SB), which is an integrating vector belonging to the family of Tc1/mariner DNA transposons, it is possible to obtain genetic modification ensuring prolonged expression in T-cells [51].

Magnani et al. conducted a study with CARCIK-CD19 (derived from a SB transposon-produced donor and differentiated CIK) in patients with B-ALL relapse after hematopoietic stem cell transplantation (HSCT). They found that, among the group of 13 evaluable patients (including 4 children), the toxicity profile was low, with no GvHD occurring. Six of the seven patients subjected to the highest doses achieved CR or CR with incomplete blood count recovery (CRi), and most reached a potent expansion of CAR-T-cells. In addition, production costs were up to 10 times lower than they were for viral processes with comparable transduction efficiency and final cell viability [52].

#### 2.1.3. Treatment by CAR-T

The patient should fulfill criteria to qualify for CAR-T-cell therapy and then undergo lymphodepletion chemotherapy to reduce the number of T lymphocytes [44,53,54]. This is advantageous because competition between the patient’s lymphocytes and newly introduced CAR-T is decreased and thus, CAR-T expression increases. CAR-T-cells recognize surface antigens independently from MHC restriction. When targeted to tumor surface antigens, CAR-T-cells proliferate and kill tumor cells upon antigen contact. Their activity is triggered by mechanisms such as continuous intense proliferation, leading to an increase in cytotoxicity, and by a boost in the secretion of interleukins, growth factors, and cytokines. 

Tisagenlecleucel is a product of anti-CD19 CAR-T-cells. It was approved as the first CAR-T gene therapy by the FDA, on 30 August 2017, for patients up to 25 years of age with refractory B-cell precursor acute lymphoblastic leukemia (BCP-ALL) or in second or later relapse [55]. This product also showed an indication of relapsed or refractory large B-lymphoma on 1 May 2018. A single-center phase 1–2a study by Maude et al. showed a CR rate of 93% in children and young adults with the disease, after CAR-T therapy [56]. Based on this, a phase 2 multicentre study was conducted, and the effectiveness turned out to be similar. In the initial phase, 92 people were enrolled. Of these, 75 received an infusion of tisagenlecleucel (CTL019). The age range of the respondents was between 3 and 25, and the median age was 11 years. The main goal of the study was to assess the safety of the tested drug and to achieve a CR on the level of 20% among patients. It was observed that 81% of these patients attained remissions within 3 months of follow-up. Forty-five patients achieved a CR, and 16 patients had a CR without a full hematological recovery. This study proved to be pivotal and led to the approval of tisagenlecleucel by the FDA (ClinicalTrials.gov number, NCT02435849) [57]. Despite the achieved remission, the presented treatment method still needs to be improved. It is associated with the questionable achievement of the prolonged survival of patients without relapse; several reasons can be found for the short effectiveness of CAR-T. Some of them are the progressive impairment of function, a decline in CAR-T-cells, and an inhibition of the BM microenvironment [58,59]. In this case, one solution may be to add allogenic hematopoietic stem cell transplantation (allo-HSCT) therapy. In a study by Davila et al., seven patients in remission received allo-HSCT, and they did not relapse within 24 months [60]. Similar results were obtained in a clinical trial by Daniel W. Lee, where patients, after such treatment, did not report a relapse within a year [61]. Another solution may be to use cells with the CD22 antigen, which persist in the blood longer than CD19 and are highly expressed in most B-ALL, and are poorly expressed in regular B cells. A study by Jing Pan noted that the CAR CD22 is capable of inducing remission even after the failure of treatment with CAR CD19; however, even after such treatment, a high relapse rate was observed. In this case, the introduction of follow-up treatment using allo-HSCT after CAR-T therapy proved to be beneficial, reducing the frequency of relapses [62]. 

Another significant problem occurs when leukemic cells stop expressing CD19 and are therefore not recognized by CD19-CAR-T-cells (the escape of the antigen from the tumor cells) [63]. Mutations in the genes could destroy the cognate epitope recognized by the anti-CD19 scFv, making the tumor cells no longer visible to CD19 CAR-T. Originally, this was thought to be due to splicing deregulation, leading to a deletion of exon 2 in the CD19 gene. However, the existence of such CD19 isoforms was only investigated following CAR-T therapy [64,65]. Subsequent studies showed that some modified CD19 isoforms, causing CAR-T escape, were not produced during therapy but existed at the time of diagnosis. During treatment, they can evolve and become a major clone of cancer cells. Such conclusions were reached by Sotillo et al. They analyzed the expression of CD19 isoforms in a cohort of 14 children with CD19+ B-ALL. By taking BM and peripheral blood samples, using semiquantitative CD19 cDNA amplification by RT-PCR, and visualizing them by agarose gel electrophoresis, three different bands were obtained. These corresponded in size to full-length CD19 (800 bp) and to exon 2 deficient isoforms detected after CAR-T treatment [64]. This makes it possible to define a set of genes encoding hitherto unknown extracellular epitopes (which are alternatively spliced in leukemia compared to normal B lymphocytes), which would allow the development of new CAR-T targeting alternative CD19 ectodomains. As a result, applying combination therapy to several epitopes may improve survival in patients with R/R B-ALL. 

CAR T-cell therapy is associated with unique and potentially severe toxicities, most particularly, cytokine release syndrome (CRS) and neurotoxicity (or “CAR-T-cell-related encephalopathy syndrome”—CRES) [44]. There are many factors that influence the intensity of CRS. It depends on the type of therapy or even the characteristics of the patients [60]. In a study conducted by Maude et al., patients experienced toxicities, and 27% of them had a severe course of CRS [61]. Teachey et al. reported that the neurotoxicity rate in children, after mild CRS, was 20% and 73% after severe CRS [66]. The fundamental mechanism of CRES is still poorly understood. The main clinical symptoms are encephalopathy followed by focal deficits and seizures. Types of focal deficits include aphasia and, less commonly, vision change or facial droop. In addition, delirium, confusion, and hallucinations may occur [61,67,68]. Most of the side effects are completely reversible, but in some cases, they can lead to death [69]. 

Therefore, a search for ways to reduce side effects has begun. Sterner et al. presented evidence that the abolition of neurotoxicity and CRS, following CAR-T use, can be achieved by neutralizing granulocyte macrophage colony stimulating factor (GM-CSF), which plays an important role in CRS mediation. The use of lenzilumab (a GM-CSF inhibitor) in this study resulted in a reduction in CRS and inflammation of the nervous system. Additionally, it intensified the proliferation of CAR-T-cells and, thus, contributed to the improvement of their therapeutic activity. The GM-CSF-reduced CAR-T-cells had, not only, a preserved therapeutic function, but they also had better survival and stronger activity against neoplastic cells. This study made it possible to reduce the main side effects of CAR-T; because of that, phase II trials with lenzilumab in this therapy are currently planned [70].

To date, the use of CARs in T-ALL has been severely limited. Although CAR-T has been shown to be effective against T-cell tumors, there are several limitations that slow the development of this therapy in T-ALL. A major obstacle is the expression of the same target antigens in both healthy and neoplastic T-cells, causing CAR-T-cells to mutually destroy each other. Another challenge is isolating normal T-cells from cancerous ones. This makes it impossible to use autologous donor T lymphocytes as a substrate for creating CAR-T based on them. Allogeneic donors can be used here, but this is associated with the likelihood of GvHD, which may be fatal. Despite this, there is still promising research on the use of CAR-T in T-ALL. CD7, the T-cell antigen, appears to be a good target that can be used in this therapy, as it is strongly expressed in T-ALL [71]. Li et al. have conducted two clinical trials to evaluate both the safety and efficacy of GC027, the allogeneic CAR-T product targeting CD7. Although only two patients with R/R T-ALL were tested, both achieved CR, and it took over a year in one of them. CRS was noticed in both patients, but GvHD was not observed [72]. As it turns out, not only can CD7 be used in the development of a new CAR-T therapy against T-ALL but so can CD1a. Such conclusions were reached by Sánchez-Martínez et al., who used it for cortical T-ALL (coT-ALL) therapy. In this subtype of leukemia, CD1a shows superficial expression, associated with developmental arrest, in the cortical stage. In this study, CD1a was found mainly in developing cortical thymocytes. It was absent in progenitor cells and T lymphocytes during ontogenesis, which reduces the risk of toxicity outside the tumor. It was also proven that CD1a-CAR-T persists for a long period of time in vivo. This indicates their safe and possible use in the case of R/R coT-ALL [73]. 

The first study on the use of CAR-T in AML appeared in 2010. Peinert et al. presented the results of the first phase of the CAR-T study in recurrent AML with Lewis (Le) -Y antigen expression. Although they did not observe severe toxicity, all patients relapsed within two years [74]. In 2013, another examination by Ritchie et al. was related to the use of second generation CD28-ζ CAR against the LeY antigen. Although only partially effective, it gave hope for the biological activity of CAR-T in the fight against AML [75]. Currently, research mainly concerns CD33 and CD123 antigens, which are largely present in AML blasts. They seem to be attractive therapeutic targets, but their presence has also been identified on healthy hematopoietic stem/progenitor cells [76]. As a result, the used CAR-T cannot distinguish between healthy and neoplastic cells, leading to myeloablation manifested by severe neutropenia, infections, and hemorrhages, leading to death. Efforts were made to create a gene that protects against myeloablation; if necessary, this would enable the elimination of T-cells within the body. Herpes simplex thymidine kinase (HSV-tk) has been successfully used, and it was able to convert a prodrug into a toxic compound that stopped the replication of genetic material, leading to cell death [77]. The use of HSV-tk has, however, been limited by its immunogenicity [78]. A better compound than HSV-tk is the co-expression of inducible caspase 9 (iCasp9) in T-cells. It connects two domains: intracellular caspase 9 (which is a pro-apoptotic protein) with human binding protein FK506 (FKBP). This combination allows conditional dimerization using a low molecular weight compound. The most important advantage of iCasp9 is low immunogenicity, as it is made up of human gene products. In addition, it retains its function even in cells with an overexpression of anti-apoptotic molecules, and the only effect in the body this causes is the elimination of transduced T lymphocytes [79]. Although iCasp9 has been tested in preclinical studies, conducted by Hoyos et al., and included in other clinical trials, there are still no conclusive data on its efficacy in CAR-T therapy [78,80]. 

A good solution seems to be to develop an antigen specific to AML cells that would not be present in any healthy cells. Although AML genomes have a low mutational load and contain few neoantigens, several have been described [81]. One of them are mutations in the metabolic enzymes IDH1 and IDH2 [82]. However, the proteins encoded by these genes are expressed inside cells, making them inaccessible to CAR-T. Therefore, it seems beneficial to develop a new CAR-T that recognizes intracellular antigens. This task was undertaken by Rafiq et al.; although they have shown that it is possible, more research is still needed on this topic [83]. 

### 2.2. Bispecific Antibodies 

An alternative approach to involving T-cells in cancer therapy is the use of antibodies, which are bispecific for CD3 in T-cells and for a surface target antigen in cancer cells. This mechanism acts independently from T-cell receptor specificity, costimulation, and antigen presentation. This new class of a bispecific T-cell-engaging (BiTE) antibody consists of two single-chain antibodies: an α-CD3 monoclonal antibody and an α-target monoclonal antibody. This construct can engage T-cells to the target neoplastic cell, subsequently activating the T-cells and causing the perforin-mediated death of the malignant cell [84]. 

The first registered BiTE in the treatment of adult and pediatric patients with BCP-ALL was blinatumomab, as the FDA granted its approval in 2018. Blinatumomab consists of an anti-CD3 arm that engages CD3-expressing T-cells and an anti-CD19 arm that binds to lymphoblasts expressing the CD19 marker [85]. In the first clinical trial, blinatumomab revealed efficacy in non-Hodgkin lymphoma (NHL), but the most important trials have been conducted in R/R ALL and in ALL with MRD. In this trial, 80% of patients became MRD-negative after the first cycle of blinatumomab, with 60% of patients remaining in CR at a median follow-up of 33 months [86,87]. Blinatumomab was approved by the FDA on 3 December 2014, after an accelerated review process in R/R Philadelphia chromosome-negative BCP-ALL [88]. 

The first results of a phase I/II study of blinatumomab in pediatric patients with R/R BCP-ALL included 49 patients in phase I and 44 patients in phase II (NCT01471782). Two of three Ph+ patients achieved CR. The primary analysis of the results indicated that, of the 70 subjects, only ±27 (39%) reached CR status, of which 14 (20%) were MRD-negative. Twenty-four patients (34%) received allo-HSCT, and 11 (16%) received consecutive treatment. The median OS was 7.5 months, with a median follow-up of 23.8 months [89]. A RIALTO study (NCT02187354) was conducted to estimate the effects of blinatumomab. Overall, 58 patients (59%) achieved CR within the first two blinatumomab cycles. Among the CR group, 39 (67%) achieved full hematologic recovery, and 46 (79%) achieved MRD response. These results demonstrate a high response rate, with blinatumomab, in pediatric patients with R/R BCP-ALL [90].

Queudeville et al. conducted retrospective analysis, of a single-center experience with blinatumomab, in 38 pediatric patients over a period of 10 years. Seventy-one percent of patients had undergone at least one HSCT prior to treatment with blinatumomab. They observed a response to blinatumomab in 13/38 patients (34%), with a median OS of 11.1 months and a relapse-free survival of 6.17 month [91]. The latest clinical trials comparing blinatumomab with standard chemotherapy in high-risk pediatric B-ALL showed promising results. An investigation conducted by Locatelli et al. included 108 patients in randomized trials. The blinatumomab group achieved event-free survival (EFS) better than the chemotherapy group. The number of deaths was in favor of the blinatumomab group, with 8 and 16 deaths in the blinatumomab and chemotherapy groups, respectively [92]. Studies conducted by Brown et al. were aimed at comparing the use of blinatumomab and chemotherapy in post-induction therapy in the first relapse of B-ALL. Studies included 208 patients (children, adolescents, and young adults; 1–27 years old); however, the randomization of risk-related subgroups had to be terminated. The results showed two-year disease-free survival (DFS) of 54.4% and 39% in the blinatumomab and chemotherapy groups, respectively (with results being not statistically significant, one-sided *p*  =  0.03). The blinatumomab group achieved a 71.3% two-year OS rate, and the chemotherapy group achieved 58.4%. The authors declared that early termination probably reduced DFS rates [93]. The main conclusion in both of these studies is that the experimental arms with blinatumomab resulted in an overall superior outcome. Blinatumomab-based therapy was also associated with a lower toxicity profile in comparison with standard chemotherapy groups [94,95]. 

Currently, numerous clinical trials are advancing, with results yet to be published. The validation of treatment protocols in children and adults with BCP-ALL is the subject of the Moscow-Berlin 2019 Pilot (NCT04723342), which includes combined treatment with the application of blinatumomab and chemotherapy in children 1–18 years old [94]. Moreover, AIEOP-BFM ALL 2017 (NCT03643276) involved new approaches in several risk-related ALL subtypes, especially in the chemotherapy-resistant or those with a high BCP-ALL relapse risk among patients up to 17 years old. Blinatumomab in this patient’s group was estimated to be a less toxic substitute for standard chemotherapy [95]. 

The Interfant-06 Protocol is investigating whether the addition of blinatumomab can improve the outcome of mixed lineage leukemia (MLL)-rearranged ALL in infants (EU Clinical Trials register 2016-004674-17) [96]. Blinatumomab is also the candidate in the bridging therapy in children and young adults (up to 25 years old), with high-risk B-ALL preceding HSCT (NCT04556084), which is assumed to improve post-HCT outcomes [97]. Furthermore, in children and young adults (1–10 years old without Down syndrome or 1–31 years old with Down syndrome) newly diagnosed with B-ALL, blinatumomab is connected with standard chemotherapy drugs (NCT03914625) to assess DFS [98]. 

Because of the rapid development of immunotherapy in hematologic malignancies, the bispecific anti-CD19/CD3 T-cell engager is tested with various immune checkpoint inhibitors (ICIs). Blinatumomab alone, or with nivolumab (anti PD-1 antibody), is being tested in children and young adults (1–31 years old) with a first relapse of CD19+ B-ALL (NCT04546399). Moreover, blinatumomab and nivolumab, with or without ipilimumab, (anti CTLA-4 antibody) have been validated for safety and dosing measures in poor-risk R/R CD19+ BCP-ALL in patients 16 years old or older (NCT02879695) [99]. BiTE targeting CD3 × CD33 are currently undergoing clinical trials as a solution to AML. Nevertheless, more studies are needed to fully evaluate these therapies [100]. 

In myeloid malignancies, a subtype of bispecific antibodies—dual affinity retargeting (DART) proteins—is used. The mechanism of action of DART is similar to BiTE; however, in their construction, an additional disulfide linker is applied. With this solution and other construction features, DART achieved improvement in the molecule stability (exceeding blinatumomab in half-life circulation time), increased the affinity to cancer cells, and lowered the affinity to T-cells (preventing non-specific T-cell activation) in comparison to a single-chain anti-CD3 antibody [101,102,103]. However, currently, no DART treatment is approved as a treatment for ALL or AML.

The remaining principles of the design and mechanism of action for DART and BiTE are similar. DART is capable of connecting two particular cells with particular molecules on their surface. In leukemic malignancies, CD3+ cells (T-cells) are activated by DART and directed to tumor cells, formatting the cytolytic synapses. Recently, CD123 has been acknowledged as a potential target in the treatment and diagnosis of AML patients. Research studies testing the MGD006 drug, currently known as flotetuzumab, have continued with preclinical and clinical trials (NCT02152956) [76,104,105,106]. Results of the first study of CD3 × CD123 DART (flotetuzumab) usage in refractory AML were recently published by Uy et al. Eighty-eight adult patients with primary induction failure or early relapse were treated with flotetuzumab, and 27% achieved CR or CR with partial hematologic recovery, where the median OS was 10.2 months. These results are correlated with standard chemotherapy protocols applied in patients in this particular stage of disease and are significantly improved with an OS threefold greater for flotetuzumab treatment [107]. Currently, clinical trials evaluate whether flotetuzumab can be used with benefits in pediatric R/R AML (NCT04158739) and in patients (>12 years old) with CD123+ hematologic malignancies (NCT04681105) and in a general expansion program (NCT04678466) [108,109,110]. DART is also tested in other leukemic malignancies, such as chronic lymphocytic leukemia (CLL), Burkitt’s lymphoma (BL), and acute monocytic leukemia (AML-M5), but these studies are limited to human cell lines [111,112]. The development of bispecific antibodies is still ongoing. Among BiTE molecules, enhancements are made in the half-life of the molecule, which results in the reduction in drug infusion quantity [113]. AMG 300 (NCT02520427), another CD3 × CD33-targeting bispecific antibody, was tested in R/R AML in adults, bringing results of anti-leukemic activity and safety in heavily pretreated patients [114]. AMG673, a CD3 × CD33 HLE bispecific antibody, also has input clinical trials in R/R AML. AMG673 (NCT03224819) demonstrated a reduction in the blast burden, but half of the patients had varying grades of CRS [115].

### 2.3. Antibody–Drug Conjugates (ADCs)

#### 2.3.1. Inotuzumab Ozogamicin

Another new type of antibody-based immunotherapy is antibody–drug conjugates (ADCs), in which a targeted antibody has anti-cancer drugs. Inotuzumab ozogamicin (InO) consists of a humanized immunoglobulin class G subtype 4 (IgG4) monoclonal antibody that allows for the delivery of cytotoxic agent N-acetyl-γ-calicheamicin dimethylhydrazide (Calich-DMH) to CD22-expressing B-cells [116,117]. It was approved by the FDA, on 17 August 2017, for the treatment of adult patients with relapsed or refractory BCP-ALL [118].

In the trial to investigate the tolerability and efficacy of InO (INO-VATE), InO monotherapy was estimated against the standard of care (SoC; intensive chemotherapy) as a first or second salvage therapy in adults with R/R BCP-ALL (NCT01564784). In the InO arm, a significantly higher proportion achieved CR/CRi (80.7% vs. 29.4%, *p* < 0.0001); among those with CR/CRi, the rate of MRD negativity was also higher (78.4% vs. 28.1%, *p* < 0.0001), and more patients proceeded directly to HSCT (41% vs. 11%) [119]. After these primary analyses, the study was continued for 2 years, and the safety and efficacy outcomes of patients were reported in 2019. They showed that, in patients with R/R BCP-ALL, InO was associated with a greater probability of CR/Cri, and it served as a bridge to HSCT. Potential veno-occlusive disease (VOD)/sinusoidal obstruction syndrome (SOS) risk factors must be considered when InO treatment decisions are being made [120]. The use of InO in 51 patients < 21 years old with R/R ALL was reported by Bhojwani et al. In this intensive pre-treated cohort, CR was achieved in 67% of patients with overt marrow disease. However, modulation of the surface CD22 was detected as a possible escape mechanism in three patients who developed a subsequent relapse after InO therapy. InO was well tolerated, with the most common side effects being cytopenia and febrile neutropenia [121]. A French multicentre pediatric retrospective study reported 12 patients ≤18 years old who had been intensively pre-treated: 5/12 with HSCT (and 8/12 with immunotherapy), blinatumomab (*n* = 6), or CAR-T (*n* = 2). Four patients were refractory to treatment. CR/CRi was observed in 8/12 patients, and two achieved MRD negativity after their first cycle of InO and are still in remission [122]. Recently, in pediatric patients (*n* = 25) with multiple R/R CD22+ ALL, functional properties of InO were confirmed in a phase 1 clinical trial (ITCC-059 study or EUDRA-CT 2016-000227-71). The overall response rate (ORR) after Course 1 was 80% of the responders, with an 84% MRD-negative CR, and the 12-month OS was 40%. Nine patients received HSCT or CAR-T-cells after InO. The authors suggested that InO was well tolerated, demonstrating antileukemic activity in intensively pretreated children with CD22+ R/R [123].

#### 2.3.2. Gemtuzumab Ozogamicin (Approved in 2017 for Pediatric >2 y.o. and Adult Cases of AML)

Gemtuzumab ozogamicin (GO) is a humanized recombinant kappa IG4 monoclonal antibody directed against N-acetyl-gamma-calicheamicin conjugated to the cytotoxin CD33 [124]. N-acetyl-gamma-calicheamicin is covalently attached to the antibody via a calicheamicin derivative linker. After binding of the ADC conjugate to tumor cells expressing the CD33 antigen, internalization of the ADC-CD33 complex follows, leading to the formed and intracellular release of N-Calich-DMH by the hydrolytic degradation of the linker. The active Calich-DMH binds to DNA, causing it to break, leading to cell cycle arrest and apoptosis [125,126]. In 2000, GO was approved by the FDA as the first monoclonal antibody conjugate for the treatment of CD33+ AML at a dose of 9 mg/m^2^, administered as an intravenous infusion, every 14 days for 28-day cycles [127]. In the study conducted by Larson et al., the authors analyzed the efficacy and safety of GO, an antibody-targeted chemotherapy for CD33-positive aAML. They reported that, when GO was administered to patients with CD33-positive AML in the first recurrence, single-agent GO induced a 26% remission rate (71/277 adult patients) with a generally acceptable safety profile [128]. An open-label dose-escalation study estimated the safety and efficacy of single-agent GO for pediatric patients with multiple relapsed or primary refractory AML. Eight of 29 (28%) patients achieved overall remission. Remissions were comparable in patients with refractory (30%) and relapsed (26%) disease. GO was relatively well tolerated at 6 mg/m^2^ for 2 doses and was equally effective in patients with R/R disease. The authors suggested that further studies, in combination with standard induction therapy for pediatric AML, are warranted [129]. Randomized phase 3 clinical trial (NCT00085709) evaluated the benefit of the addition of GO to standard induction and postconsolidation therapy in patients with AML. Among those who achieved CR, the five-year relapse-free survival rate was 43% in the DA + GO (daunorubicin + gemtuzumab ozogamicin) group and 42% in the DA group (*p* = 0.40). The five-year OS rate was 46% in the DA + GO group and 50% in the DA group (*p* = 0.85). DFS was not improved with postconsolidation GO (HR, 1.48; *p* = 0.97). In this study, the addition of GO to induction or postconsolidation therapy failed to show improvement in the CR rate, DFS, or OS [130]. Gamis et al. have presented data of Children’s Oncology Group (COG) AAML0531, showing a reduced relapse risk when GO was given in upfront therapy (32.8% vs. 41.3%), but treatment-related mortality has increased (8.6% vs. 5.9%). GO significantly improved EFS (3 years: 53.1% vs. 46.9%) but not OS (3 years: 69.4% vs. 65.4%). Remission was not improved (88% vs. 85%; *p* = 0.15) [131]. A study conducted by Niktoreh et al. indicates an urgent need for uniform prospective studies on patients with R/R AML. The probability of 4-year OS was 18 ± 5% in all patients, 27 ± 7% in patients with HSCT, and 0% in patients without it (*p* < 0.0001). The administration of GO on a patient-specific, use basis was frequently considered in their study group and proved to be effective for bridging very advanced AML to HSCT in children [132]. Recently, Pollard et al. investigated the impact of GO on survival in pediatric patients with KMT2A-rearranged (KMT2A-r) AML enrolled in the COG trial AAML0531 (NCT01407757). Of the 1,022 patients enrolled, 21% had KMT2A-r AML. Five-year EFS and OS from study entry were 38% and 58%, respectively. EFS was superior with GO treatment (48% with GO vs. 29% without, *p* = 0.003), although OS was comparable (63% vs. 53%, *p* = 0.054). The GO benefit was observed in both high-risk and non-high-risk KMT2A-r subsets. For patients who underwent HSCT, prior GO exposure was associated with decreased relapse. In multivariable analysis, GO was independently associated with improved EFS, improved DFS, and reduced RR. The authors recommended that future clinical trials should study CD33-targeted agents, in combination with HSCT, for pediatric KMT2A-r AML [133].

## 3. Future Perspectives in the Treatment of ALL or AML

### 3.1. Monoclonal Antibodies

#### 3.1.1. Daratumumab

Daratumumab is a human immunoglobulin G1 kappa (IgG1κ) monoclonal antibody [134]. Its role is to bind to a specific epitope on CD38-expressing cells, thus leading to their apoptosis. This process can occur through several mechanisms, such as antibody-dependent cell-mediated cytotoxicity, complement-dependent cytotoxicity, or antibody-dependent cellular phagocytosis [135,136,137]. In turn, CD38 is a type II transmembrane glycoprotein, whose role is to regulate the cytoplasmic flow of Ca^2+^ ions and mediate signal transduction in myeloid and lymphoid cells [138,139]. Moreover, it has been shown that CD38 is highly expressed in myeloma cells, compared to its low expression in normal lymphoid and myeloid cells [140]. 

This discovery led to the FDA approval of daratumumab in 2015 as the first monoclonal antibody for the treatment of patients with multiple myeloma, who had received at least three prior therapies [141]. Currently, there are promising studies that may lead to the use of this monoclonal antibody in pediatric T-ALL as well. This assumption is dictated by the fact that the presence of CD38 on blasts in children and young adults with T-ALL has been proven. In addition, it was shown that the expression of CD38 was maintained in the examined patients at a constant level, even after multivariate chemotherapy [142]. This result of the study by Bride et al. led to further research in this direction. Vogiatzi et al.’s trial showed that the use of daratumumab contributed to the elimination of MRD in a preclinical model of T-ALL in children. Moreover, the prolongation of survival in the daratumumab group turned out to be statistically significant, compared to the untreated control group [143]. Such evidence supports the conclusion that daratumumab may prove to be a powerful therapeutic option in patients with T-ALL, for which further research is needed. We look forward to the results of a currently ongoing study, which is a phase II trial of daratumumab, in addition to standard chemotherapy, for use in children and young adults (≤30 years old) with R/R ALL from both B and T-cells (ClinicalTrials.gov identifier: NCT03384654) [144].

#### 3.1.2. Alemtuzumab

Alemtuzumab, as with daratumumab, is a human IgG1κ monoclonal antibody [145]. The mechanism of action of this antibody is based on binding to CD52, which leads to cell death by antibody-dependent cellular cytotoxicity (ADCC) and through complement-dependent cell lysis (CDC) [146]. The presence of CD52 has been demonstrated in many cells, such as B and T lymphocytes, where it is strongly expressed, and monocytes and macrophages, which include the lower levels of CD52 [147]. Moreover, a high expression of CD52 (also known as the CAMPATH-1 antigen) was demonstrated on most malignant lymphoid cells, which resulted in the first (accelerated) registration of alemtuzumab, on 7 May 2001, by the FDA for the treatment of chronic B-cell lymphocytic leukemia (B-CLL) [148]. Conversion from accelerated to regular approval of alemtuzumab to B-CLL, by the FDA, took place on 19 September 2007 [149]. In addition, the effectiveness of alemtuzumab has also been proven in relapsing multiple sclerosis, which led to its approval by the FDA in 2014 [145,150,151].

As CD52 was expressed in acute leukemia blasts, it was suspected that alemtuzumab might prove beneficial in the treatment of ALL or AML [152]. One of the studies conducted in this direction was the trial by Angiolillo et al., which concerned the use of alemtuzumab in children and young adults with R/R CD52+ ALL. Out of 13 people participating in the study, only one achieved CR, which did not encourage similar research [153]. In addition to trying to treat ALL patients with the direct involvement of alemtuzumab, the antibody has also been attempted to improve other therapies. Due to stem cell transplantation from HLA-compliant donor siblings, more than 60% of pediatric ALL patients can be cured. However, less than 30% of them will have compatible siblings. In this case, an alternative donor transplantation can be used, but it is associated with a higher risk of GvHD. Since adding alemtuzumab to an alternative donor transplant causes a reduction in T-cell counts, there is a theory that this may ultimately reduce the risk of GvHD. To verify this, Kennedy-Nasser et al. decided to conduct a study on a group of 83 children with ALL. They compared two groups of patients: HLA-matching stem cell transplantation and donor alternative (AD) transplantation with alemtuzumab. In the presented results, they showed that, in both groups, the DFS and the number of relapses were at a similar level. Moreover, the incidence of GvHD was also similar. The only great difference between the two groups was treatment-related mortality, where it was higher in the AD group. However, this may be a consequence of the prolonged immunosuppression associated with alemtuzumab. In the AD group, a significantly higher rate of infection reactivation was observed, and six patients died from viral infection [154]. In another study, the results were similar. Rao et al. also concluded that the use of alemtuzumab, as part of a conditioning regimen, prior to allo-HSCT (unrelated donor) in pediatric patients contributed to the reduction in severe GvHD. Contrary to the study presented earlier, no increased risk of life-threatening infections was noted here. This supports the view that strict infection surveillance and the appropriate use of prophylactic measures can play a significant role in preventing severe post-transplant infections [155]. In addition, an increased incidence of infection-related deaths after allo-HSCT in children was also noted in a study by Lindsay et al., where the use of alemtuzumab was also reported as one of the reasons [156]. The presented results prove that it is possible to reduce the risk of GvHD by using alemtuzumab in alternative transplant recipients. However, it is imperative to develop better antiviral therapies to reduce patient mortality.

#### 3.1.3. Rituximab

Rituximab is a humanized murine monoclonal antibody targeting the CD20 antigen, and the combination of these components induces cell death [157]. Its effect is based on complement-dependent cytotoxicity and antibody-dependent cell-mediated cytotoxicity. Moreover, it participates in the induction of apoptosis and the sensitization of cancer cells to chemotherapy [158].

The first approval of rituximab by the FDA was in 1997 to treat R/R CD20-positive B-cell NHL [159]. Since then, many studies have led to its use in other diseases, such as CLL, rheumatoid arthritis, granulomatosis with polyangiitis, microscopic polyangiitis, and pemphigus vulgaris [160,161,162,163,164]. As CD20 expression is associated with poor prognosis in adult ALL, Thomas et al. decided to check the impact of the incorporation of rituximab in the treatment for adults with BCP-ALL and showed that the inclusion of this monoclonal antibody in hyper-CVAD (cyclophosphamide, vincristine, adriamycin, and dexamethasone) therapy in CD20+ patients contributed to a one-year DFS rate of 100%. For comparison, this rate, in the group treated without rituximab, was 49% [165]. Jeha et al. have conducted a related study on a group of 353 children with BCP-ALL, 169 of whom showed CD20 expression. In contrast to the adult study, it was shown here that the current expression of CD20 was associated with better patient outcomes. However, there was still a question of whether the inclusion of monoclonal therapy in the treatment could lead to better patient outcomes [166]. Another study was performed to investigate the response rate and toxicity to rituximab in combination with chemotherapy. It was conducted by Griffin et al. and included pediatric patients with R/R B-cell NHL and mature B-ALL. Toxicity (although common after rituximab infusion) was manageable, and the CR/partial response ratio for the entire group of subjects was 12/20, which was quite encouraging for further research. Only two of the examined patients suffered from B-ALL, which proves that, in the context of this disease, the trial result was quite unreliable [167]. In turn, in a study by Rigaud et al., the survival of children and adolescents with mature lymphoma/B-cell leukemia remained low after relapse, and no significant improvement was seen with rituximab [168]. Although the presented studies do not prove the effectiveness of rituximab in childhood ALL, they do not deny it either. It has been shown that the expression of CD20 in BCP-ALL blasts may be increased after the use of steroids [169]. This suggests that an attempt to use a rituximab + steroid combination therapy in BCP-ALL, by increasing CD20 expression, could increase the effectiveness of anti-CD20 therapy and thus improve patient outcomes. However, in order to confirm this theory, appropriate studies should be carried out. Despite the lack of evidence to support the efficacy of rituximab in pediatric ALL, its role in the other diseases mentioned above is unquestionable. For this reason, it was decided to develop new anti-CD20 monoclonal antibodies, such as ofatumumab and obinutuzumab, on which research is still ongoing [170].

#### 3.1.4. Ofatumumab 

Ofatumumab is an Ig1 fully human monoclonal anti-CD20 antibody that is linked to the membrane-proximal epitope in CD20+ cells [171]. Ofatumumab is characterized by low dissociation rates and strong complement-dependent cytotoxicity, leading to tumor cell lysis [172]. In 2009, ofatumumab was approved by the FDA in the treatment of patients with refractory CLL [173]. A phase II clinical trial (NCT01363128), combining chemotherapy with ofatumumab in patients with ALL or lymphoblastic lymphoma at all ages, is awaiting results [174]. Similar studies have been conducted on patients with R/R ALL (NCT03136146) [175]. Moreover, combinations of ofatumumab and other therapies (blinatumomab, inotuzumab ozogamicin, and chemotherapy) are under investigation. The new study (NCT02877303) involves 14-year-old patients (and older), and it will determine the efficiency of the presented therapeutic solutions in patients with B-ALL [176].

#### 3.1.5. Moxetumomab Pasudotox

Moxetumomab pasudotox is a recombinant immunotoxin targeting CD22. It consists of the Fv fragment of the murine anti-CD22 monoclonal antibody RFB4, linked to a truncated form of the pseudomonas exotoxin A, PE38 [177,178]. Other names that this immunotoxin may appear under are CAT-8015, HA22, or moxetumomab [179]. It was first approved in 2018, by the USA FDA, for the treatment of adults with R/R hairy cell leukemia (HCL), who had received at least two prior systemic therapies, including treatment with a purine nucleoside analogue [180]. Despite the discontinuation of the use of moxetumomab pasudotox in precursor leukemia/lymphoblastic lymphoma, NHL, and CLL, studies with this immunotoxin in other diseases are still ongoing [177]. One of the studies on the use of moxetumomab in BCP-ALL was conducted by Mussai et al. Analyzing the therapeutic effectiveness of HA22 against B-ALL blasts in pediatric patients suggested its cytotoxic impact and provided additional support for another ongoing clinical trial [181]. The phase I trial by Wayne et al. (NCT00659425) included patients aged 6 months to 25 years, with multiple relapse or chemotherapy-refractory ALL, who had received ≥1 standard and 1 salvage regimen of allo-HSCT. Of the 47 patients assessed, objective response rates were observed in 32%. Additionally, 5 of the 11 people who achieved composite CRs became MRD-negative [182,183]. This study provided evidence that moxetumomab pasudotox is active against CD22+ ALL in children and can overcome resistance to chemotherapy. This result prompted the researchers to conduct a phase II study to further evaluate the efficacy of moxetumomab. Thirty-two patients, with a median age of 10, were enrolled in the study. Among the patients who received the study drug and were assessed for its effectiveness, an objective response rate was reported in 28.6%, and 10.7% achieved MRD-positive morphological CR. Though 21.4% of patients achieved disease stabilization, progression occurred in 39.3% [184]. The results obtained did not support the transition to phase III, but moxetumomab showed some activity in patients with B-ALL. Further studies on this topic may be considered.

#### 3.1.6. Combotox

Combotox is a combination of two murine IgG1 monoclonal antibodies, anti-CD19 (HD37) and anti-CD22 (RFB4), linked together by a deglycosylated ricin A chain (dgRTA) to produce an immunotoxin [185]. HD37-dgRTA and RFB4-dgRTA have been shown to act separately, but their ability to kill tumor cells has also been shown to be additive, indicating their greater efficacy in this combination [186,187,188]. In order to evaluate the safety and efficacy of combotox, Herrera et al. decided to conduct an appropriate study. It was a phase I study involving a group of pediatric patients with R/R ALL. Of the 17 patients, aged 1–16 years, three patients experienced CR, six experienced a >95% blast count decrease in peripheral blood, and one patient experienced a 75% blast count decrease. The obtained results showed the effectiveness of combotox. However, the reduction in the number of peripheral blasts was generally short-lived [185].

As there is evidence that efficacy can be improved by adding cytotoxic agents to immunotoxins, a study was conducted using this combination [189,190]. Barta et al. conducted a study to evaluate the efficacy of combotox + cytarabine (a cytotoxic agent commonly used in the treatment of ALL) in a murine advanced BCP-ALL cell xenograft model [187]. In their results, they showed that the combination of both low and high doses of combotox and cytotoxic agent cytarabine (Ara-C) contributes to the prolongation of the median survival. Moreover, they showed that the sequential administration of Ara-C and combotox is preferable to simultaneous administration. Based on this, a phase I trial was conducted, evaluating this combination in adults with R/R B-ALL (NCT01408160) [191].

#### 3.1.7. Denintuzumab Mafodotin (SGN-CD19A)

Denintuzumab mafodotin is the drug conjugate that combines a humanized anti-CD19 antibody with the anti-mitotic agent monomethyl auristatin F (MMAF). MMAF is an anti-tubulin agent inhibiting the cell division process [192]. Results of a phase I clinical trial with this drug showed safety and clinical activity in adult and young adult patients with R/R B-ALL, B-cell lymphoma, and Burkitt leukemia/lymphoma (NCT01786096). In the weekly dosing scheme, 19% (of 32 patients) achieved CR; once every three weeks, 35% (of 23 patients) achieved CR [193,194]. Another safe study, covering patients with B-cell lymphoma (including BCP-ALL) 12 years old or older, was completed (NCT01786135); however, the results are yet to come [195]. Jones et al. proved that SGN-CD19A is an active therapy against pediatric ALL-patient-derived xenografts. However, the denintuzumab mafodotin preclinical activity levels did not outperform those achieved with vincristine as a single agent [196].

#### 3.1.8. Loncastuximab Tesirine (ADCT-402)

Loncastuximab tesirine is another antibody drug that combines monoclonal antibody against CD19+ cells conjugated to a pyrrolobenzodiazepine (PBD) dimer—SG3199 [197]. The mechanism of action of SG3199 is to create cytotoxic interstrand DNA cross-links. These cross-links are known for their non-distorting nature, which enables SG3199 to erase slowly proliferating cells [198]. Phase 1 studies of ADCT-402 included patients 12 years old or older with previously pretreated R/R B-ALL. Thirty-five patients were enrolled, and three of them achieved CR. Summary results were weaker in response rate than other novel therapies (including blinatumomab, inotuzumab ozogamicin, and tisagenlecleucel). The study was terminated by the sponsor, and the authors could not match the criteria, so a recommended dose of ADCT-402 was not estimated [199]. Further studies are required to determine which patients can benefit from this therapy.

#### 3.1.9. Camidanlumab Tesirine (Cami-T or ADCT-301)

Camidanlumab tesirine is an ADC delivering the SG3199 dimer, and it is conjugated with a human monoclonal antibody against interleukin-2 receptor alpha chain (IL2RA). It is also called CD25. The IL2RA is highly expressed in the surface of leukemic cells (ALL) with a prognostic value for those patients [200]. Studies conducted by Goldberg et al. on 35 adult patients (34 with AML and 1 with ALL) tested the limits of Camidanlumab tesirine dosage and tolerability. Despite this trial being terminated, they observed CR in two patients and good toleration of this drug [201]. Currently, ADCT-301 is tested in children and adults with Hodgkins lymphoma (NCT04052997) and young adults with AML, myelodysplastic syndrome (MDS), and myeloproliferative neoplasm (MPN) (NCT04639024) [202,203].

#### 3.1.10. Coltuximab Ravtansine (SAR3419)

Coltuximab ravtansine combines huB4, being the humanized anti-CD-19 (anti-B4) antibody, with a derived maytansine (DM4), which is a microtubule-affecting cytotoxic element [204,205]. This drug was tested in patients with diagnosed B-ALL older than 16 years old (NCT01440179); however, the study was cancelled due to the moderate activity among these patients, compared to other available therapies [206]. Preclinical trials conducted on xenografts from BCP-ALL and infant MLL showed SAR3419 activity in the induction of remission [207]. These results were confirmed with an insignificant clinical response in patients with R/R ALL [208]. However, these studies were the origin of the new ADC invention, the huB4-DGN462. HuB4-DGN462 consists of DGN462, which is an indolinobenzodiazepine pseudodimer with anti-tumor activity, and the HuB4 part is known to be from SAR3419. This new ADC presents enhanced anti-tumor activity, even with low levels of CD19 biomarkers, which will be examined in further studies [209]. 

#### 3.1.11. Epratuzumab

Epratuzumab was originally designed for NHL and leukemia, but it is also being used in Sjögren’s disease and in systemic lupus erythematosus (SLE) [210]. The Pediatric Oncology Group has decided to conduct a series of studies evaluating the effectiveness of epratuzumab in the context of relapsed BCP-ALL in children. Raetz et al. focused on determining tolerability, serum epratuzumab levels, and its efficacy, administered alone and in combination with reinduction chemotherapy in children with relapsed CD22+ BCP-ALL. Fifteen patients, ranging in age from 2 to 21 years old, were assessed at the end of 1 block (during which two patients died of infection, and one was dropped from the protocol). Nine patients achieved CR, and seven of them had no detectable MRD. In addition, epratuzumab has been shown to be properly tolerated. The most common side effects, such as fever and rigor, occurred during the first infusion and responded favorably to steroids and meperidine [211]. Such an encouraging result of the study contributed to the second part of the trial. This time, an attempt was made to determine whether the addition of epratuzumab to chemotherapy may contribute to the improvement of the results of the second CR in BCP-ALL with early BM relapse [212]. Initially, epratuzumab was administered once a week (Cohort B1); however, due to its shorter half-life in children with ALL, the dosage was increased to twice a week (Cohort B2). It was shown that 65% of patients with B1 and 66% with B2 achieved second remission (CR2). However, compared to the historical control group, treated with chemotherapy alone (COG AALL01P2), the CR2 ratio did not improve. However, this was different with the rate of MRD. Of the B1 and B2 groups, 42% of patients were MRD-negative, compared to 25% with chemotherapy alone [212,213]. It was shown that, in the next phase of the study, epratuzumab, in combination with reinduction chemotherapy, is well tolerated in children and adolescents with early relapses of CD22+ B-ALL. Therefore, randomized phase III trials are currently underway to provide better insight into the action of this antibody (www.clinicaltrials.gov; identifier: NCT01802814). The same applies for other therapeutic approaches summarized in our review (Table 3).

**Figure 1:**
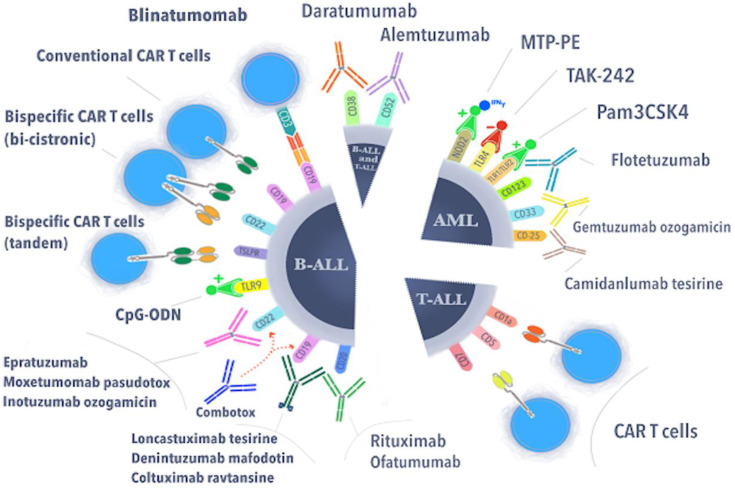
Current therapeutic approaches in the treatment of pediatric cases of acute leukemias.

As can be seen from the cited studies, for many years, attempts have been made to investigate the relationship of CART, antibody drug conjugates, and monoclonal antibodies with pediatric cases of leukemia, such as AML and ALL (Figure 1).

### 3.2. Immune Checkpoint Inhibitors

#### 3.2.1. CTLA-4 Antibodies

Cytotoxic T-lymphocyte-associated protein 4 (CTLA-4) belongs to the immunoglobulin superfamily, playing a costimulatory role expressed by activated T-cells. CTLA-4 is responsible for the downregulation of immune response (lowering IL-2 levels), also functioning as an immune checkpoint expressed on the surface of activated T-cells and in regulatory T-cells (Tregs) [214,215]. Similar to the T-cell costimulatory CD28, CTLA-4 is highly competitive to the CD80 (B7-1) and CD86 (B7-2) ligands, which can be found on the surface of Antigen Presenting Cells (APC). Moreover, CD28, in contrast to CTLA-4, acts as a stimulating factor mediated by Phosphoinositide 3-kinases (PI3K) and Protein kinase B (PKB), also known as AKT, but with weaker affinity and avidity to their ligand [216]. Cancer cells are known to have increased B7-1 levels, which inhibit immune system response [217]. Hence, blocking the inhibition of T-cell response is the principal of the CTLA-4 antigen’s mechanism of action, resulting in the elimination of cancer cells [218]. CTLA-4-neutralizing antibodies are now approved for the treatment of advanced melanoma and are in development for treating other cancers as well.

Ipilimumab is a fully human monoclonal antibody (IgG1), blocking CTLA-4 to promote anti-tumor immunity [219]. Ipilimumab has shown activity in patients with metastatic melanoma when it has been used as a monotherapy in phase 2 studies [220]. Since then, the CTLA-4 inhibitor (frequently combined with nivolumab) has been tested and approved in the treatment of renal cell carcinoma, metastatic colorectal cancer, untreated unresectable malignant pleural mesothelioma, and others.

Bashey et al. have assessed the safety and preliminary efficacy of a neutralizing, human anti-CTLA4 monoclonal antibody, ipilimumab, in stimulating the graft-versus-malignancy (GVM) effect after allo-HCT. Twenty-nine patients, with malignancies that were recurrent or progressive after allo-HCT, received ipilimumab as a single infusion at dose cohorts between 0.1 and 3.0 mg/kg. Three patients with lymphoid malignancy developed objective disease responses following ipilimumab: CR in two patients with Hodgkin disease and partial remission (PR) in a patient with refractory mantle cell lymphoma [221]. Davids et al. used an immune checkpoint blockade, established by CTLA-4, with ipilimumab (10 mg/kg) to restore antitumor reactivity through a graft-versus-tumor (GVT) effect in hematologic patients (*n* = 28, 12 with AML, and 1 with ALL) after allo-HSCT. Results were encouraging, with five patients gaining CR to this treatment and two with a partial response. OS in 12 months was 49% [222]. In leukemias, there are currently few clinical trials focusing on ipilimumab usage. One of them examines the combined therapy of blinatumomab and nivolumab, with or without ipilimumab, in patients with R/R CD19+ BCP-ALL (NCT02879695) [99]. Furthermore, Penter et al. proved that ipilimumab can enhance the graft-versus-leukemia (GVL) effect and reinvigorate T-cells. Three of 44 patients reached CR, and three reached transient response statuses [223]. Treatment of relapsed AML with ipilimumab and decitabine is now in clinical trials (NCT02890329), but results have not been published yet [224].

CTLA-4 antigens are known for the exacerbation or activation of autoimmune diseases and for the induction of diverse adverse effects, the most common of which are hypophysitis, acute kidney injury, arthritis, and colitis [225,226]. Thus, immune-related adverse effects should be managed with precaution and evidence-based medicine [227]. There is an urgent need to determine all regulatory functions of CTLA-4-related immunological pathways. Current medicine is focused on combining CTLA-4 antigens with other ICIs and various therapies.

#### 3.2.2. Programmed Cell Death Protein (PD-1, CD279)

A member of the T-cell regulators family is the programmed cell death protein 1 (PD-1, CD279), expressed in activated T-cells, B-cells, natural killer T (NKT) cells, and activated macrophages. After connecting to its ligands, which are PD-L1 (B7-H1) and PD-L2 (B7-DC), present on the surface of APC (macrophages and dendritic cells), PD-1 functions, as a promotor of apoptosis of T-cells and as an inhibitor to T-cell response [228,229]. In the mechanism of evacuation from T-cell toxicity, tumor cells often express PD-L1 on their surface, which hampers the immune system [230,231]. Furthermore, PD-L1 expression is responsible for Treg expansion, which results in immune system suppression. Tregs are expected to promote AML blast growth by IL-10 and IL-35 secretion [232]. Hence, currently, the approach to the PD-1/PD-L1 complex is managed in both ways: by blocking the PD-1 or by blocking the PD-L1, with PD-1 antibodies being superior over PD-L1 in OS rates among cancer patients [227].

#### 3.2.3. PD-1 Inhibitors

The first registered PD-1 targeting drug was pembrolizumab (MK-3475, lambrolizumab), which is a humanized monoclonal IgG4 anti-PD-1 antibody. In the beginning, it was registered in advanced melanoma, after achieving auspicious results in a group of patients, after unsuccessful treatment with ipilimumab [233,234]. First trials of pembrolizumab (NCT02767934) failed in improving outcomes of patients with T-ALL and B-ALL with measurable MRD. A study conducted by Cassaday et al. achieved a median OS value of only 12.7 months, so the trial was terminated [235].

Another PD-1 immune checkpoint blocker, nivolumab (BMS-936558, ONO-4538), is also a fully human IgG4 antibody. At first, it was tested in refractory solid tumors, followed by clinical trials on patients with advanced squamous non-small cell lung cancer [236,237]. In 2014, nivolumab was approved by the FDA for the treatment of patients with advanced melanoma [238]. Currently, it is being tested in adult patients with a high risk of AML relapse (NCT02532231). First results have showed that the blockade of the PD-1 checkpoint does not lower the estimated time of CR in these patients, so more optimistic data are not expected [239]. Another phase II clinical trial (NCT04546399) included patients with a first relapse of CD19+ B-ALL (1–31 years old), with treatment options of nivolumab combined with blinatumomab or blinatumomab alone [240]. A trial with R/R CD19+ BCP-ALL in children and young adult patients, using a combination of pembrolizumab and blinatumomab, was withdrawn (NCT03605589) [241]. The safety and tolerability of a combination of blinatumomab, ipilimumab, and nivolumab therapy will mainly be evaluated as a treatment of patients (16 years and older) diagnosed with poor-risk R/R CD19+ BCP-ALL or CD19+ mixed phenotype acute leukemia (MPAL) (NCT02879695) [99]. Studies, conducted by Kamphorst et al. on mice models, showed that a blockade of PD-1 may not be sufficient for the improvement in response from CD8+ T-cells. The co-stimulation of CD28 is required in PD-1 inhibitor therapy to achieve exceptional results. Therefore, the usage of CD28/CD80 and the CD86 pathway may increase clinical outcomes in various cancer regimens [242]. Moreover, it was found that PD-1 levels are at their highest levels in relapsed B-ALL, which encourages a benefit from the PD-1 blockade in such patients [243]. In the BM of AML patients, PD-1 and TIM-3 levels were elevated, presumably hampering T-cell response [244]. PD-1 inhibitor-related adverse effects (irAEs) of nivolumab include skin irritation (rash) and pulmonary and hepatic symptoms, and those of pembrolizumab include endocrinopathies, rash, and diarrhea [245].

#### 3.2.4. PD-L1 Inhibitors

Atezolizumab (MPDL3280A) is the first PD-L1 inhibitor approved by the FDA in the treatment of urothelial carcinoma, as a result of studies conducted by Rosenberg et al. [246]. It is a human monoclonal IgG1 antibody targeting this immune checkpoint [247]. In AML, atezolizumab was tested with the combination of immunomodulatory agents (Guadecitabine) in adults with R/R, treatment-naive, and chemotherapy-resistant patients (NCT02892318) [248]. However, its results have still not been published. There are no clinical trials with PD-L1 antibodies as a therapeutic approach in childhood leukemias. Durvalumab (MEDI4736), another human PD-L1 antibody, is registered as a treatment of patients with locally advanced or metastatic urothelial carcinoma [249]. It was first tested in patients with solid tumors, achieving clinical activity [250]. Later, durvalumab improved response rates in patients with advanced urothelial bladder cancer, resulting in FDA approval in this group of patients [251]. This drug was tested, in combination with azacitidine, as a front-line treatment for chemotherapy-unfit AML patients, bringing the expected results of an absence of any increase in PD-L1 surface expression. Remembering that azacitidine is known for increasing PD-L1 levels, a blockade of PD-L1 is a bright regimen solution [252]. Atezolizumab-treated patients developed pulmonary symptoms and diarrhea, while adverse effects of durvalumab included endocrinopathies and pulmonary symptoms [245].

### 3.3. Other Targets for Immune Checkpoint Inhibitors

#### 3.3.1. Anti-CD47 Antibodies

CD-47 acts as a signaling receptor for thrombospondin 1 and the counter-receptor for the signal-regulatory protein alpha (SIRPα). All functions of this protein and its ligands are yet to be discovered [253]. In general, CD47 acts as a phagocytosis inhibitor signal, commonly referred to as the “do not eat me” message. A crucial role is also played by the SIRPα present in macrophages and dendritic cells. A high expression of CD47 protein in cancer cells correlates with a poor prognosis for patients, due to the gained ability of evasion from macrophages and dendritic cells. Magrolimab (Hu5F9-G4) is a humanized monoclonal antibody targeting CD47 present on macrophages and dendritic cells, enhancing CD47-dependent phagocytosis [254,255]. After achieving promising preclinical results with in vivo and in vitro studies in AML cells, Hu5F9-G4 was forwarded into clinical trials [256]. Due to the fact that CD47 is expressed in red blood cells, the impact of anti-47 monoclonal antibody Hu5F9-G4 on blood parameters was examined (NCT02678338). The obtained results state that haemoglobin levels declined during this treatment [257]. Another phase 1b study covered magrolimab with azacytidine in adult patients with R/R AML (NCT03248479) and showed an objective response, particularly in seven of eight patients with TP53 mutation [258]. Magrolimab-related adverse effects mainly include dermatitis acneiform, dry skin, fatigue, and infusion reactions [259]. 

#### 3.3.2. Anti T-Cell Immunoglobulin and a Mucin-Domain Containing-3 (Tim-3) Antibody

**Figure 2:**
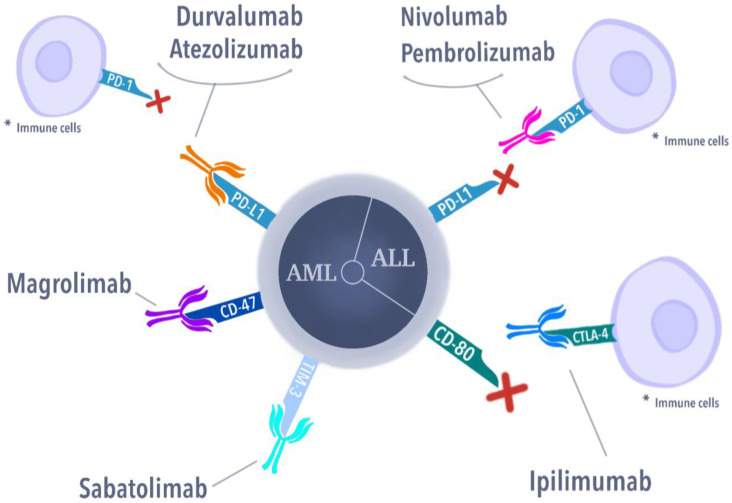
Immune checkpoint inhibitors are pediatric cases of acute leukemias. * Immune cells are Antigen Presenting Cells (B lymphocytes, dendritic cells, and macrophages) for PD-1+ cells. CTLA-4 is present in activated T-cells.

Tim-3 regulates the secretion of TNF and interferon γ (IFN-γ), which results in the inhibition of Th1 cell response. TIM-3 is also expressed in monocytes, macrophages, dendritic cells, tumor infiltrating lymphocytes, and leukemic stem cells and blasts (without healthy hematopoietic stem cells (HSCs)) [260,261,262]. There are several ligands for the TIM-3 molecule: galectin 9 (Gal9), high mobility group protein B1, phosphatidylserine, and CEACAM1 [263]. Dama et al. discovered that, in AML patients, TIM-3 and Gal9 levels were significantly higher in patients with treatment failure, which means that, in those patients, blocking this pathway may improve therapeutic results, with a clinical outcome of CR [264]. Sabatolimab (MBG453) is a humanized IgG4 antibody that targets TIM-3. It was tested in 35 patients with newly diagnosed AML and resulted in an ORR of 41.2%, with an estimated 12 months of progression-free survival at a rate of 44% [262]. Currently, sabatolimab is being tested in AML patients after allo-HSCT with present MRD. This study (NCT04623216) covers patients from 12 to 99 years old, and results are yet to come [265]. Moreover, the combination of sabatolimab with blocked PD-1 (PDR001) and decatibine is an active phase I study (NCT03066648), connecting various ICI therapy targets [266]. A frequently occurring adverse effect was fatigue, which was present in 9% of 133 patients [267]. ICIs, which are in use in pediatric cases of hematological malignancies, are shown below (Figure 2).

### 3.4. Pattern Recognition Receptors (PRRs) 

Pattern recognition receptors (PRRs) are germline-encoded proteins capable of recognizing pathogen-related molecules [268]. They are expressed mainly by macrophages, monocytes, neutrophils, dendritic cells, and epithelial cells, i.e., by cells of the innate immune system [269]. Their role is to detect two classes of these molecules: PAMPs (pathogen-associated molecular patterns) and DAMPs (danger-associated molecular patterns). When PRRs interact with PAMPs, a complete signaling cascade is initiated, leading to the transcription of genes involved in inflammatory responses. These genes encode cytokines, chemokines, type I interferons, proteins involved in the modulation of PRR activity, and many others. Their expression depends on the type of activated PRR. As a consequence, pro-inflammatory compounds, dependent on cells such as dendritic cells, are secreted [270]. This is the foundation for the differentiation of antigen-specific T-cells. In addition, PRRs also contribute to the activation of programmed cell death, including apoptosis, which, in turn, affects the release of DAMPs. In the next phase, DAMPs stimulate the cells of the immune system, which also occurs with the participation of PRRs. Such a mechanism leads to the creation of a kind of positive feedback, where each subsequent stage stimulates the previous one. This increases the patient’s immune response and thus strengthens his defense mechanisms [268]. To date, several classes of PRRs have been discovered. They can be classified according to their function, location, or specificity for concrete ligands. If their location division is assumed, it is possible to distinguish two subgroups: cytoplasmic and related to the cell membrane. In this case, the cytoplasmic PRRs include NOD-like receptors (NLRs) and RIG-I-like receptors (RLRs). On the other hand, Toll-like receptors (TLRs) and type C lectin receptors (CTLs) are classified as membrane-bound [271].

#### 3.4.1. Toll-like Receptors (TLRs)

Toll-like receptors are a class of type 1 single-pass transmembrane glycoproteins. They recognize ligands outside the cell and contribute to the initiation of a pro-inflammatory response. They contain an extracellular domain, composed of leucin-rich repeats and the cytoplasmic domain of the Toll/interleukin-1 receptor (TIR), which is the analogue of interleukin 1 receptors (IL-1R) [272]. The TIR domain tightly controls all signals passing through the TLR. It owes its function to the presence of adapter proteins, which include myeloid differentiation factor 88 (MyD88), TIR domain containing adapter inducing interferon-β (TRIF), the TRIF-related adapter molecule (TRAM), and TIR domain-containing adaptor protein/MyD88-adaptor-like (TIRAP/Mal). The presence of several subtypes of the TIR domain ensures specificity for TLR-dependent signaling pathways [273]. TLR signaling can take two directions: the MyD88-dependent pathway and the TRIF-dependent pathway. In the first pathway, when TLR activation occurs, MyD88 recruit interleukin-1 receptor-associated kinase 4 (IRAK-4) to TLR [274]. IRAK-4 then mediates the phosphorylation of IRAK-1, which interacts with the TNF R-associated factor 6 (TRAF6) E3 ubiquitin ligase. TRAF6 is involved in the activation of transforming growth factor beta-activated kinase 1 (TAK1). TAK1 activates the nuclear k-enhancer factor of the light chain of activated B cells (NF-κB) and the mitogen activated protein kinase (MAPK) pathways. As a consequence, pro-inflammatory cytokines, such as interleukin-1 (IL-1), IL-6, IL-8, and TNFα, are induced. Involved in this path is the TIRAP/Mal adapter, which mediates between MyD88 and TLR2 or TLR4 [273,275].

Although most TLRs work via the featured MyD88, this is not the case with TLR-3. This receptor, instead of MyD88, uses the adaptor protein TRIF [276]. When TRIF binds to TRAF3, it activates IKK-associated kinases TBK1 and IKKε. This leads to the inclusion of the regulatory factor interferon-3 (IRF3) and stimulation of the production of type I interferons (IFNs) [277]. Additionally, TRIF interacting with TRAF6 leads to the activation of NF-κB and MAPK, independently of MyD88 [278]. TLR4 is also worth mentioning, because it can work via TRIF and MyD88. Moreover, for its TRIF-dependent pathway, TRAM is specific. By combining TLR4 with TRIF, it is involved in the organization of the inflammatory response under the influence of a specific pathogen [273,277,279]. 

There are many subgroups of TLRs. It is currently known that 10 of these (TLR1-TLR10) are found in humans [280]. The fundamental classification of TLRs is based on their ability to be activated by various ligands. In addition, they may contain various adapters and be located either on the surface of the cell or inside it. With this division, the TLRs present on the cell surface include TLR1-2, TLR4-6, and TLR10, and those located in the inner compartments of the cell are TLR3 and TLR7-9. The ligands principally include lipid-derived molecules such as lipopeptides (TLR1-2, TLR6, TLR10), nucleic acid fragments (TLR3, TLR7-9), lipopolysaccharides (TLR4), and flagellin (TLR5). TLR3 recognizes the double-stranded RNA (dsRNA) of viruses, while single-stranded RNA (ssRNA) is detected by TLR7-8 [281,282,283,284]. TLRs can bind to more than one type of ligand, such as TLR4, which, in addition to lipopolysaccharides, also reacts to heparan sulfate fragments, fibrinogen, or nickel [273,285]. All these features translate into their specificity for the detected microorganisms. Accordingly, TLR1-2 and TLR4-6 are typical for bacterial infection, TLR3 and TLR7-8 are for viral infection, and TLR9 is for both [286]. A summary of TLRs, their dependent pathways, locations, and sample ligands is provided in Table 4.

The importance of TLRs during infection is unmatched. However, for many years, scientists have wondered whether the potential of TLRs can also be used to fight other diseases. At the same time, more data on inflammation and recurrent infections, in patients with hematological malignancies, have begun to appear, showing an association with TLRs. More than 10 years ago, it was suggested that TLRs may condition hematopoiesis by acting from the level of HSCs [287]. A study by Sioud et al. confirmed that TLR4, TLR7, and TLR8 can be expressed by human hematopoietic BM CD34+ progenitor cells. Additionally, it was noted that TLR7/8 signaling influenced further differentiation of CD34+ cells [288]. It is known that a hallmark of acute leukemias is the uncontrolled production of the hematopoietic precursor cells of the myeloid or lymphoid series in the BM [289]. This suggests the possibility that primitive TLR-expressing cells are the beginning of unstable lineages. Moreover, a study by Eriksson et al. shows that TLR1 expression is much more potent in the immature CD34+ and CD38- cells of AML patients, as compared to normal cells [290]. This was in line with the elevated levels of TLR1 mRNA in patients with MDS [291].

Additionally, Eriksson evaluated the effect that both the agonist and antagonist of TLR1 would have on leukemia stem cells. After applying an inhibitory effect on TLR1, a strong decrease in the number of leukemic cells was observed, which confirmed that this receptor is important for AML activity. On the other hand, after the use of Pam3CSK4, which is a specific TLR1-2 agonist, an increase in the survival of leukemic cells was observed. However, further forced TLR1/TLR2 signaling led to the differentiation of leukemic cells and thus, a reduction in the leukemia burden [290]. This proves that further studies based on the inhibition or forced activation of TLR1 in AML could prove beneficial in achieving satisfactory clinical effects.

Apart from TLR1-2, TLR4 may also play a significant role in the disease activity of AML. As in the case of the previous receptors, it was assumed that the use of a TLR4 inhibitor may slow the activity of AML. To do this, a study by Baakhlagh et al. was conducted. TAK-242 was used there as a TLR4 inhibitor. The obtained results showed that the applied inhibitor not only stopped the proliferation of all cell lines of leukemia, but also changed the distribution of their cell cycle. Additionally, it was noticed that, in AML patients with poor prognosis, there is a much higher expression of TLR4, MyD88, and NF-кB mRNA [292]. In light of this evidence, it seems advantageous to continue research on TLR4 and its inhibitor, as it may be a good strategy for the treatment of TLR4-expressing AML in the future. Aref et al. investigated the impact of TLR2 and TLR4 polymorphisms on the survival of patients with AML and their susceptibility to severe infections [293]. They demonstrated that, in AML patients with TLR2 Arg753Gln with an AG genotype and an A allele, TLR4 Asp299Gly with a CT genotype and a C allele, and TLR4 Thr399Ile with an AG genotype and an A allele, pneumonia and sepsis were more frequent than in patients with other genotypes and alleles. These results are in line with an earlier study by Schnetzke [294]. At the same time, it was observed that patients with the TLR2 (Arg753Gln) GG genotype had the shortest OS, which differed from the Schnetzke results. This study showed that, in these patients, TLR polymorphism translates into both their risk of infection and survival. However, more research is needed on this point [293].

Sánchez-Cuaxosp et al. decided to conduct the first study on pediatric ALL patients. It showed that, in children with this leukemia, there is a reduced expression of TLR1, TLR3, TLR4, TLR7, and TLR9 compared to the control group. Moreover, they considered patients with different phenotypes, such as Pro-B, Pre-B, and B- and T-ALL. They noticed that the lowest expression of TLR4 and TLR7 was found in patients with Pro-B and B-ALL subtypes. As emphasized, this could have been influenced by many factors, such as the type of cells or the type of stimulus. In addition, it is believed that the age of the patient may also be an influence. Thus, there is still no relevant data to unequivocally point to the cause of this variability in TLR levels. At the same time, the presented study suggests that the decreased expression of TLRs, in ALL patients, may partially explain the decreased anti-tumor response in this group of children [295]. 

Other strategies to prevent relapse in ALL employ synthetic single-stranded oligonucleotides, containing unmethylated cytosine and guanine (CpG-ODN) motifs, acting as TLR9 agonists [296]. Many years ago, it was noticed that CpG-ODN (GNKG168) stimulates the formation of cytokines produced by pediatric ALL cells in vitro. As a result, it increased the allogeneic response of Th1 cells against this leukemia [297]. The Therapeutic Advances in Childhood Leukemia and Lymphoma decided to conduct their own study. Their goal was to investigate the effect of GNKG168 on immunomodulatory molecules in MDR-positive children with ALL and those in remission. They compared the expression levels of 608 genes before and eight days after using GNKG168 in patients. In their results, they focused on the eight markers that showed the greatest difference before and after the study. They observed an increase in the level of H-RAS mRNA and promyelocytic leukemia protein, but a decrease in single Ig and TIR domain containing (SIGIRR, IL1R8) interleukin 1 receptor 1 (IL1RL1, ST2), CC motif chemokine receptor 8 (CCR8), interleukin 7 R (IL7R), cluster of differentiation 8B (CD8B), and cluster of differentiation 3 (CD3D). Summarizing, it can be noted that the described increase in expression concerned genes involved in increasing B and T lymphocyte responses. On the other hand, the decrease in expression was related to genes and involved enhancing the immune response to tumorigenesis. It is possible that the tested GNKG168 act as checkpoint inhibitors, as it caused a decrease in SIGIRR, IL1RL1, CCR8, and IL7R, which are checkpoint signals. In order to make clear conclusions, more studies are needed on larger groups of patients [298]. Recently, the use of a properly constructed vaccine has started to be considered as well. As vaccines induce and potentiate the patient’s T-cells, this one could target leukemia-associated antigens. The past year has led to a human trial using a vaccine based on TLRs. It was assumed that this vaccine could induce an anti-leukemic immune response. To test this, patients in AML remission, with a high risk of relapse, were inoculated with mature TLR7/8 RNA-loaded dendritic cells encoding two antigens associated with this leukemia: WT1 and PRAME. The obtained results showed that the administered vaccine elicited a favorable local inflammatory response and led to the proliferation of T CD8+ and T CD4+ in peripheral blood, which was clinically advantageous. In addition, its administration was found to be safe for patients, and the response was better suited to their younger age [299]. Although this is a promising strategy for the future therapy of AML, much more research, both on children and adults, is still needed on the subject. Additionally, it might be beneficial to combine mature 7/8 TLR cells with, e.g., checkpoint inhibitors, which would contribute to an additional enhancement of the immune response of patients.

#### 3.4.2. NOD-Like Receptors (NLRs)

Nucleotide-binding oligomerization domain (NOD), as with receptors (NLRs), belong to the group of cytosolic PRRs. As receptors located inside the cell, they are involved in the detection of intracellular pathogens and endogenous products generated during tissue damage [300]. So far, 22 types of NLRs in humans have been described [271,301]. They are characterized by a common organization of domains, centrally containing NATCH, which is necessary for the activation of the NLR by ATP. In addition, there is an N-terminal effector domain to mediate signal transduction and a C-terminal region, having a varying number of leucine-rich repeat (LRR) motifs domains, involved in molecular pattern recognition [301,302]. Depending on their N-terminal region, four subgroups can be distinguished among NLRs: NLRA (the acid transactivation domain), NLRC (the domain of caspase activation and recruitment), NLRB (the inhibitory repeat-like baculovirus domain), and NLRP (the N-terminal pyrin effector domain) [301].

NLRC includes nucleotide-binding oligomerization domain 1 (NOD1), nucleotide-binding oligomerization domain 2 (NOD2), and domains classified in this group due to their homology and phylogenetic relationships (NLRC3, NLRC4, NLRC5, and NLRX1) [303]. The role of NOD1 and NOD2 is to detect bacterial particles produced during the synthesis or degradation of peptidoglycan. More specifically, NOD1 detects the γ-D-glutamyl-meso-diaminopimelinic acid dipeptide (iE-DAP), a component of the peptidoglycan found in most Gram-negative bacteria and specific Gram-positive bacteria. In contrast, NOD2 recognizes muramyl dipeptide (MDP), which is present in virtually all types of peptidoglycans [304,305]. Ligand recognition, by the LRR of NOD1 and NOD2, leads to activation of the serine-threonine kinase (RICK). This kinase activates the TAK1 kinase, leading in turn to the degradation of the IκBα (nuclear factor kappa B inhibitor). As a result, NF-κB translocation to the nucleus takes place, where appropriate NF-κB-dependent genes can be transcribed. NOD1 and NOD2, in addition to NF-κB, also activate MAP kinase. As a result, the NF-κB and MAPK work together to increase the expression of pro-inflammatory factors [302].

NOD1 and NOD2 have been minimally tested for acute leukemias, but this does not mean that these receptors cannot be successfully used in the future in the treatment of patients with acute leukemia. Similar to the TLRs, it has been shown that NOD2 is expressed by human BM CD34+ cells [306]. This suggests that the activation of this receptor and the subsequent inflammatory response may play a role in AML. To verify this, Buteyn et al. evaluated the effect of NOD2 receptors on AML. They decided to use phosphatidylethanolamine muramyl ripeptide (MTP-PE), which is a synthetic and less pyrogenic derivative of MDP, a NOD2 ligand. However, they found that MTP-PE alone was unable to produce the expected anti-tumor effects. As a result, they decided to combine it with IFN-γ. This combination led to satisfactory results, as it led to a significant apoptosis of AML blasts in a caspase-1-dependent manner. It was reported that MTP-PE, in tandem with IFN-γ, caused the release of pro-inflammatory cytokines, such as TNF-α and IL-1β, and supported the maturation of NK cells, both in vitro and in a murine adoptive transfer model. In addition, it was shown that this combination reduced the severity of the disease and increased the survival rate of the tested mice [307]. Thus, this study showed that NOD2 agonists may have a beneficial therapeutic effect on AML.

Apart from the role that NODs can play in leukemia, their polymorphism also seems interesting. Undoubtedly, many AML patients have a reduced number of neutrophils at the time of diagnosis [308]. Additionally, the use of induction chemotherapy leads to the prolongation of the neutropenia phase. As some of the sites of NOD2 expression are neutrophils [309], Yomade et al. decided to check whether the NOD2 gene polymorphism would translate into the frequency of infections in AML patients after intensive induction chemotherapy. They focused on an analysis of the three most common polymorphisms: two missense mutations (Arg702Trp and Gly908Arg) and the frameshift mutation (Leu100fsinsC). After analyzing the results of 131 patients with AML, they concluded that patients without any of the listed polymorphisms have a much lower likelihood of developing enteritis or mucositis. Conversely, patients with these polymorphisms were more likely to have *Streptococcus* spp. detected in the bloodstream. However, it is worth adding that the presence of these mutations did not affect the incidence of complications, such as pneumonia or sepsis [310]. The presented research shows that there is a possibility of linking NOD2 polymorphisms to the frequency of infections in AML patients, but more studies are needed to confirm this.

## 4. Conclusions

In recent years, tremendous advances have been made in the treatment of childhood acute leukemias. Currently, functioning therapies have reached such a high level that their further intensification does not make sense, as they are associated with increased toxicity. Therefore, methods using the immune system have been developed to treat patients in whom standard therapies have failed. Based on advances in genetics, it has become possible to understand the molecular mechanisms of leukemia pathogenesis. Comprehensive genome-wide sequencing and integration analyses identified new leukemia subtypes with different prognostics and therapeutic factors. Moreover, it is now possible to detect antibodies present in leukemia cells, such as CD52, CD38, CD33, CD20, CD25, CD19, and CD22, and target them with new antibody–drug conjugates. The development of new technologies makes immunotherapy more common, and cheaper, for patients and can also be used to treat other diseases. 

In the last few years, there has been a breakthrough in the treatment of ALL with CAR-Ts. This is evidenced not only by the drug approved for this purpose by the FDA, but also by the amount of research conducted on this subject. As shown, attempts have been made for years to develop a satisfactory use of CAR-Ts in AML therapy, and although many barriers have been encountered along this path, further clinical trials are still ongoing. In the case of ICIs, our knowledge of their function, expression, and targeted therapies has expanded significantly and may become a milestone in modern medicine and a hope for patients. The known checkpoints from other diseases are very likely to be adapted to the treatment of leukemia in adults and then in children, resulting in even better treatment outcomes. Investigations about the relationship between TLRs and leukemia such as AML and ALL are still ongoing. However, we still do not have any conclusive data on their exact function in the diseases under consideration. It is possible that we will finally be able to fully interpret their role in cancers and thus, improve patient survival. Although the lines of research on NOD in acute leukemia seem similar to those of TLRs, they are much smaller and at an earlier stage. However, this does not exclude the possibility of their future use in AML or ALL therapy. For that to happen, a substantial amount of research has to be done. 

In our work, we focused on showing the latest therapeutic possibilities of curing patients with selected acute leukemias. Starting with presenting therapies whose effectiveness has already been proven, we proceeded to summarize the future prospects for the treatment of AML and ALL. We have chosen children as the main group because they are patients that show great potential for the use of the above-mentioned methods of treatment.

In addition, we believe that treatment based on targeted therapies, in which a drug is individually selected for a patient, represents the future of medicine.

With this in mind, we can obtain an advantage in the fight against hematological neoplasms, contributing to the improvement of the conditions of patients.

## Figures and Tables

**Table 1:** Genetic subgroups of B-cell acute lymphoblastic leukemia in children.

Genetic Subgroup	Frequency in BCP-ALL	Characteristics	Prognosis	Therapeutic Approach	References
Hyperdiploidy	25–30%	51–67 chromosomes; 3 to 5 years old	excellent	MRD-based reduction in intensity treatment; condensin-complex members, AURKB, or the spindle assembly checkpoint	[4,5]
*ETV6::RUNX1* t(12;21)(p13.2;q22.1)	20–25%	Commonly occurring with the deletion of non-rearranged *ETV6* allele, the deletion of *PAX5*, and the deletion of 6q	excellent	Conventional chemotherapy with reduced intensity	[6]
High hypodiploidy	2–3%	40–44 chromosomes, often occurring with dic(9;20) or *ETV6::RUNX1*	poor	MRD risk-stratified therapy	[7]
Near haploidy	<1%	24–31 chromosomes; associated with mutations of *NF1*, *FLT3*, *NRAS*, *KRAS*, *MAPK1*, and *PTPN11* and the deletion/mutation of *IKZF3*	poor	BCL-2 inhibitors, immunotherapy, phosphoinositide 3-kinase (PI3K) inhibitors	[8]
Low haploidy	2%	32–32 chromosomes; the deletion of *IKZF2*, *RB1*, *CDKN2A*/*CDKN2B*, and *TP53* mutations	poor	BCL-2 inhibitors, immunotherapy, phosphoinositide 3-kinase (PI3K) inhibitors	[8]
*TCF3::PBX1* t(1;19)(q23;p13)	5–6%	higher WBC at diagnosis; increased CNS relapse cases	poor	dasatinib and ponatinib	[9]
*KMT2A::AFF1* t(4;11)(q21;q23)	4%	pro-B (CD10-) immunophenotype, expression of myeloid markers	very poor	Intensification of treatment; DOT1L inhibitors, menin inhibitors, proteasome inhibitors, histone deacetylase inhibitors, BCL-2 inhibitors; CAR-T therapy	[10,11,12]
*KMT2A-*rearranged (11q23)	2–3%	common in infant ALL (80%); <100 different partner genes	poor	DOT1L inhibitors, menin inhibitors, proteasome inhibitors, histone deacetylase inhibitors, BCL-2 inhibitors; CAR-T therapy	[12]
*TCF3::HLF* t(17;19)(	<0.7%	associated with the expression of stem cells and myeloid markers, alterations of *PAX5,* *VPREB1* and the Ras signaling pathway	very poor	BCL-2 inhibitors (venetoclax), immunologic therapies, Aurora A kinase inhibitors	[13]
*BCR::ABL1* t(9;22)(q34.1;q11.2)	2–3%	increases with age; associated with IKZF1, PAX5, CDKN2A/B deletions, and hypodiploidy	poor	improved outcome when chemotherapy combined with TKI: imatinib, dasatinib, ponatinib	[14]
iAMP21	1–2%	additional copies of a region of chromosome 21 that includes *RUNX1*, and it can be associated with older age (median, 9 years) and low white blood cell counts	poor	intensive treatment improves outcome	[15]
Ph-like or BCR-ABL1-like ALL	10–15%	increases with age; frequently harbor alterations of *IKZF1;* alterations include: JAK-STAT (*CRLF2* rearrangement (*P2RY8::CRLF2* and *IGH::CRLF2*; *CRLF2* F232C mutation) ABL1 class fusions (*ABL1*, *ABL2*, *PDGFRB*, *CSF1R*, *PDGFRA*, and *LYN*) fewer common fusions (*FLT3*, *FGFR1*, *NTRK3*, and *PTK2B*)	poor	ABL1 inhibitors, BCL-2 inhibitors, blinatumomab, inotuzumab, and CAR-T cells	[16,17,18]

MRD: minimal residual disease; AURKB: Aurora Kinase B; BCL-2; PI3K: Phosphoinositide3-kinase; WBC: white blood cells; CNS: central nervous system; DOT1L: DOT1 similar to histone lysine methylotransferase; CAR-T: chimeric antigen receptor T-cells; ALL: acute lymphoblastic leukemia; TKI: tyrosine kinase inhibitor.

**Table 2:** Genetic subgroups of acute myeloid leukemia in children.

Genetic Subgroup	Frequency	Characteristics	Prognosis	References
*RUNX1::RUNX1T1* t(8;21)(q22;q22)	10–12%	FAB M2, blasts with single and thin Auer rods, median age 8 years; CBF AML; standard risk group; almost 90% of patients achieve complete remission with chemotherapy alone; dasatinib (targeting KIT kinase); GO for relapsed patients	very good	[20,21,22,23,24,25]
*CBFB::MYH11* inv(16)(p13.1q22) or t(16;16)(p13.1;q22)	8–10%	FAB M4eo, median age 9 years; core binding factor (CBF) AML; standard risk group; almost 90% of patients achieve complete remission with chemotherapy alone; dasatinib (targeting KIT kinase); gemtuzumab ozogamicin (GO) for relapsed patients	very good	[20,21,22,23,24,25]
*PML::RARA* t(15;17)(q24.1;q21.2)	5–10%	FAB M3, median age 7 years (1–18 years); acute promyelocytic leukemia (APL); standard risk group; ATRA, ATO treatment	very good	[20,21,22,23,24,26,27]
*KMT2A-* rearranged (11q23)	16–21%	FAB M4 and M5, infant, median age 7 years (1–18 years) KMT2A with multiple partners; hypomethylating agents, DOT1L inhibitors, Menin-KMT2A protein–protein interaction inhibitors, protein interaction inhibitors	poor or intermediate	[10,11,20,21,22,23,24,25,28,29,30]
*KMT2A::MLLT3* t(9;11)(p22;q23)	6–9%	identified in 40% of *KMT2Ar* AML cases	intermediate	
*KMT2A::MLLT1* t(11;19)(q23;p13.3)	1%	identified in 7% of *KMT2Ar* AML cases	intermediate	
*KMT2A::ELL* t(11;19)(q23;p13.1)	1–2%	identified in 7% of *KMT2Ar* AML cases	poor	
*KMT2A::MLLT10* t(10;11)(p12;q23)	2–3%	identified in 6% of *KMT2Ar* AML cases	poor	
*KMT2A::MLLT4* t(6;11)(q27;q23)	1–2%	identified in 8% of *KMT2Ar* AML cases	poor	
*NUP98::NSD1* t(5;11)(q35;p15)	3–4%	FAB M4 and M5; median age 10.4 years; 10% strong association with *FLT3*-ITD	poor	[20,21,22,23,24,25,31,32,33]
*NUP98::KMD5A* t(11;12)(p15;p13)	1–2%	30% of FAB M7 (AMKL); median age 3.2 years		
*MNX1::ETV6* t(7;12)(q36;p13)	1%	Only infants (4% of infants); 3-year EFS below 24%; KAT inhibitors, C646, I-CBP112, CCS1477	poor	[20,21,22,23,24,25]
*DEK::NUP214* t(6;9)(p22;q34)	1–4%	FAB M2 and M4; median age 12 years, no infant; association with *FLT3*-ITD; benefit from HSCT in first CR	poor	[20,21,22,23,24,25]
*CBFA2T3::GLIS2* inv(16)(p13.3;q24.3)	2–3%	FAB M7 (AMKL); infants; median age 1.5 years; 20% of non-DS-AMKL; high rates of relapse, and dismal survival; GLI inhibitors (GANT61); the AURKA inhibitor alisertib (MLN8237)	very poor	[20,21,22,23,24,25,31,32]
*BCR::ABL1* t(9;22)(q34;q11)	1%	sensitivity to TKI	poor	[20,21,22,23,24,25]
*KAT6A::CREBBP* t(8;16)(p11;p13)	<1%	FAB M4 and M5; infants; spontaneous remission has been observed	intermediate	[20,21,22,23,24,25,34]
*RBM15::MKL1* t(1;22)(p13;q13)	<1%	FAB M7 (AMKL); median age 0.7 years; 14% of non-DS-AMKL	intermediate	[20,21,22,23,24,25,31,32]
*PICALM::MLLT10* t(10;11)(p12;q14)	<1%	Extramedullary disease, CD7+, older children	intermediate	[20,21,22,23,24,25]
*EVI1(MECOM)* inv(3)(q21q26.2)/ t(3;3)(q21;q26.2)	<2%	Median age 3 years; secondary abnormality monosomy 7	poor	[20,21,22,23,24,25]
*NPM1::MLF1* t(3;5)(q25;q35)	<0.5%	FAB M2, M4, and M5; median age 3.5 years	intermediate	[35]
*FUS::ERG* t(16;21)(p11;q22)	<0.4%	Median age 8.5 years	poor	[36]
*RUNX1::CBFA2T3* t(16;21)(q24;q22)	<0.2%	FAB M1/M2, t-AML; median age 6.8 years	unknown	[36]
Monosomy 7/del(7q)	3%	median age 7 years; are considered candidates for allo-HSCT in first complete remission (CR)	poor	[20,21,22,23,24,25]
Monosomy 5/del(5q)	1–2%	FAB M0; median age 12.5 years; 10-year OS 30–40% 10-year EFS 30%; are considered candidates for allo-HSCT in first CR	poor	[20,21,22,23,24,25]
Trisomy 8	10–14%	median age 10 years	unknown	[37]
Hyperdiploidy (48–65 chromosomes)	11%	FAB M7 (AMKL); infants; median age 2 years	no significance	[38]

CBF: core binding factor; KIT: receptor tyrosine kinas; GO: gemtuzumab ozogamicin; APL: acute promyelocytic leukemia; ATRA: All-trans-retinoic acid; ATO: arsenic tiroxide; KMT2A: Lysine (K)-specific methyltransferase 2A; DOT1L: Disruptor of telomeric silencing 1-like; *KMT2Ar: KMT2A-rearranged*; AMKL: Acute megakaryoblastic leukemia; EFS: Event free survival; HSCT: Hematopoietic stem cell transplantation; CR: Complete remission; non-DS-AMKL: Non-Down Syndrome Acute megakaryoblastic leukemia; GLI: Glioma associated; AURKA: Aurora KInase A; TKI: Tyrosine kinase inhibitor; Allo-HSCT: Allogenic hematopoietic stem cell transplantation.

**Table 3:** Antibody–drug conjugates and monoclonal antibodies used in the treatment of pediatric cases of acute lymphoblastic leukemia and acute myeloid leukemia.

Targeted Molecule	Drug Name (Symbol)	Mechanism of Action	Pediatric Hematological Malignancy
CD19	Denintuzumab mafodotin (SGN-CD19A)	Inhibition of cell division in CD19+ cells	ALL/B-ALL
	Coltuximab Ravtansine	Disruption of microtubules, impeding cell cycle	B-ALL
	Loncastuximab tesirine (ADCT-402)	Creating cytotoxic interstrand DNA cross-links in CD19+ cells	B-ALL
CD25	Camidanlumab tesirine (ADCT-301)	Creating cytotoxic interstrand DNA cross-links in CD25+ cells	ALL/AML
CD20	Rituximab	Complement-dependent cytotoxicity, antibody-dependent cell-mediated cytotoxicity	B-ALL
	Ofatumumab	Complement-dependent cytotoxicity	B-ALL
CD22	Epratuzumab	Modulate the activation of B lymphocytes	relapse B-ALL
	Inotuzumab ozogamicin	Breaking double-stranded DNA, hampering the cell cycle (at the G2/M phase)	B-ALL
	Moxetumomab pasudotox	Targeting in CD22 causes cytotoxicity relative to B-ALL blasts	B-ALL
CD33	Gemtuzumab ozogamicin	Blocking cell cycle resulting in tumor cell death	AML
CD52	Alemtuzumab	Binding to CD52 leads to cell death by ADCC and through CDC	risk reduction in GVHD in patients with ALL after an alternative donor transplantation
CD38	Daratumumab	Its role is to bind to a specific epitope on CD38-expressing cells, thus leading to their apoptosis.	T-ALL

**Table 4:** Toll-like receptors with their pathways, ligands, and localizations.

Toll-Like Receptor	Dependent Pathway	Ligand	Localization
TLR1	MyD88	Lipopeptides	Cell surface
TLR2	MyD88, TIRAP, MAL	Lipopeptides	Cell surface
TLR3	TRIF	Nucelic acid fragments	Inner cell compartments
TLR4	MyD88, TIRAP, TRAM	Lipopolisaccharydes	Cell surface
TLR5	MyD88	Flagellin	Cell surface
TLR6	MyD88	Lipopeptides	Cell surface
TLR7	MyD88	Nucelic acid fragments	Inner cell compartments
TLR8	MyD88	Nucelic acid fragments	Inner cell compartments
TLR9	MyD88	Nucelic acid fragments	Inner cell compartments
TLR10	MyD88	Lipopeptides	Cell surface

## Data Availability

No new data were created or analyzed in this study. Data sharing is not applicable to this article.
